# Antireflective grassy surface on glass substrates with self-masked dry etching

**DOI:** 10.1186/1556-276X-8-505

**Published:** 2013-12-01

**Authors:** Young Min Song, Gyeong Cheol Park, Eun Kyu Kang, Chan Il Yeo, Yong Tak Lee

**Affiliations:** 1 School of Information and Mechatronics, Gwangju Institute of Science and Technology, 1 Oryong-dong, Buk-gu, Gwangju 500-712, Republic of Korea; 2 Department of Electronic Engineering, Pusan National University, 2 Busandaehak-ro 63beong-gil, Geumjeong-gu, Busan 609-735, Republic of Korea

**Keywords:** Antireflection, Glass, Subwavelength structure, Self-masked dry etching, Hydrophilicity

## Abstract

Although recently developed bio-inspired nanostructures exhibit superior optic performance, their practical applications are limited due to cost issues. We present highly transparent glasses with grassy surface fabricated with self-masked dry etch process. Simultaneously generated nanoclusters during reactive ion etch process with simple gas mixture (i.e., CF_4_/O_2_) enables lithography-free, one-step nanostructure fabrication. The resulting grassy surfaces, composed of tapered subwavelength structures, exhibit antireflective (AR) properties in 300 to 1,800-nm wavelength ranges as well as improved hydrophilicity for antifogging. Rigorous coupled-wave analysis calculation provides design guidelines for AR surface on glass substrates.

## Background

Antireflective (AR) coatings/structures are needed for most of existing optical components and optoelectronic devices, ranging from glasses, polymers, and fibers to solar cells, photodetectors, light-emitting diodes, and laser diodes, to remove undesired optical loss and improve optical performance [1-3]. For advanced AR properties compared to the conventional AR coatings (i.e., very low reflection at broad wavelength ranges and large incident angles), subwavelength structures (SWSs) with tapered profile, which is inspired by insect's eye, have been developed [4-6]. Because the SWSs have only zeroth diffraction order, it is possible to control the effective refractive index by changing the curvature of SWSs. From the theoretical understanding of SWSs and precise control of geometries (i.e., period, height, shape and packing density), improved AR performances of various materials and their device applications have been recently reported [7-9].

There are a variety of fabrication processes for AR SWSs, such as electron-beam or laser interference lithography, nanoimprint lithography, nanosphere or colloid formation, metal nanoparticles, and Langmuir-Blodgett assembly [5,6,8-15]. However, these techniques are still expensive, time consuming, and sophisticated, which block the penetration of commercial market. In case of transparent glasses, although the importance of AR structures for improvement of optical efficiency, the cost issues have hindered the use of AR structures in applications such as photovoltaics and optoelectronics. In this letter, we present a simple, fast, and cost-effective method for fabricating AR grassy surfaces composed of tapered SWSs on glass substrates. Reactive ion etch (RIE) process of glasses with gas mixture of CF_4_ and O_2_ generates nanoclusters that can be used as an etch mask. Control of etch conditions provides optimal AR performance in the visible wavelength ranges.

## Methods

### Design and fabrication

**Figure 1:**
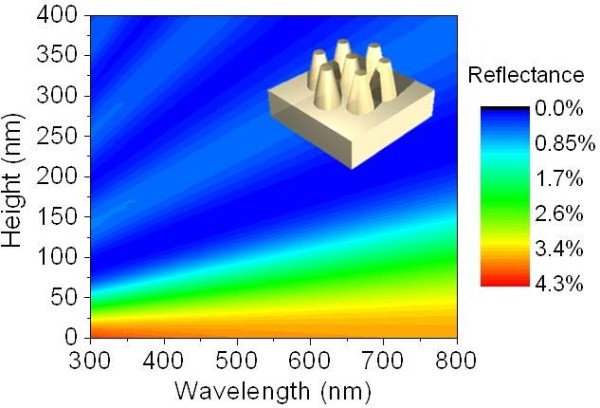
**Contour plot of calculated reflectance of tapered SWSs as a function of height and wavelength.** Inset indicates a calculated model.

According to theoretical analysis, the subwavelength structures (SWSs) with high aspect ratio (i.e., fine period and tall height) and continuous tapered shape from the air to the substrate show the widest bandwidth and almost omnidirectional AR properties [1]. However, fine tuning of geometry increases process complexity and costs. It is essential to find the optimal geometry based on the theoretical calculation to obtain a reasonable AR performance. Figure 1 shows the color map of reflectance of the SWSs on glass substrates as a function of height (0 to 400 nm) and wavelength (300 to 800 nm), calculated by a rigorous coupled-wave analysis method [16]. A model was designed in hexagonal lattices of 100 nm, which is small enough to satisfy zeroth order condition (Λ <<*λ*). The dispersion of glass material (BoroFloat 33, Schott, Louisville, KY, USA) was taken into account in this calculation. The apex diameter was set to 50% of the base diameter. The flat surface (height = 0 nm) of glass substrate shows the reflectance of approximately 4% as expected. This reflectance rapidly goes down to 1% as the height increases from 0 to 150 nm. This is available only when the index difference is not quite big. For semiconductor materials such as silicon and GaAs, the height should be at least >300 nm to have broadband antireflection characteristics. In this study, the SWSs with height of approximately 150 nm were selected as a target value to maintain a low surface reflection.

**Figure 2:**
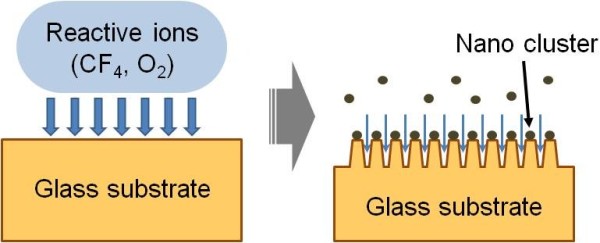
Schematic illustration of grassy surface formation with self-masked dry etching.

Uniform and high-density grassy surfaces were prepared by plasma etching in an RIE system with gas mixture of CF_4_ (40 sccm) and O_2_ (10 sccm), as illustrated in Figure 2. First, borosilicate glass substrates (2 × 2 cm^2^), which is commonly used as an optic component in various fields, were cleaned with acetone, isopropyl alcohol, and deionized (DI) water and loaded into the chamber. Afterward, simultaneous process of self-masking and reactive etching of the unmasked area for grassy surface formation was done in one step. The process pressure was 50 mTorr and the RF power was varied from 50 to 150 W. The fabricated samples were cleaned with DI water and analyzed using a field-emission scanning electron microscope (FE-SEM, S-4700, Hitachi, Ltd., Tokyo, Japan). The transmittance spectra of the samples were measured with a UV–Vis-NIR spectrophotometer (Cary 500, Varian, Inc., Palo Alto, CA, USA) in the wavelength range of 300 to 1,800 nm.

## Results and discussion

**Figure 3:**
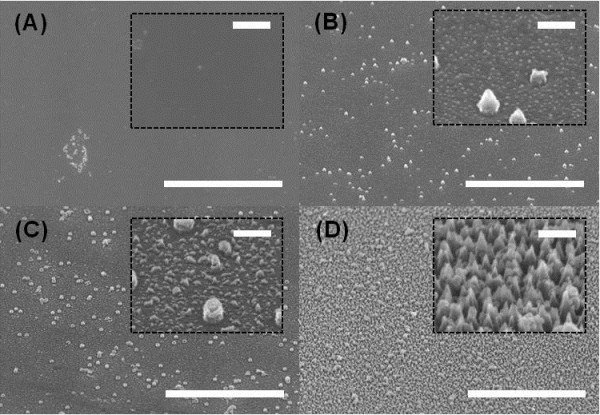
**SEM images of etched surface of glass substrates.** SEM images of etched surface of glass substrates after dry etching in RIE for 3 min with RF power of **(A)** 150, **(B)** 100, **(C)** 75, and **(D)** 50 W, respectively. The insets show the magnified images. Scale bars of main figures and insets correspond to 5 μm and 300 nm, respectively.

Figure 3 shows tilted-view SEM images of the etched surface with different RF powers. The morphology of etched surfaces drastically changed with the RF power, as exhibited in Figure 3. Grassy etched surfaces observed at low bias powers of 100 W indicate the existence of nanoscale masks, while a smoother surface was obtained at a higher bias power of 150 W. This tendency can be found in other literature [17]. It is believed that during the RIE etching with low RF power, nonvolatile nanoscale clusters are formed from the reaction of glass and reactive ions, and these clusters are uniformly distributed over the entire surface. Meanwhile, CF_4_ and O_2_ plasma are responsible for the etching of exposed surface. At 50 W RF power, the resulting grassy surface has tapered SWSs with diameter of approximately 100 nm.

**Figure 4:**
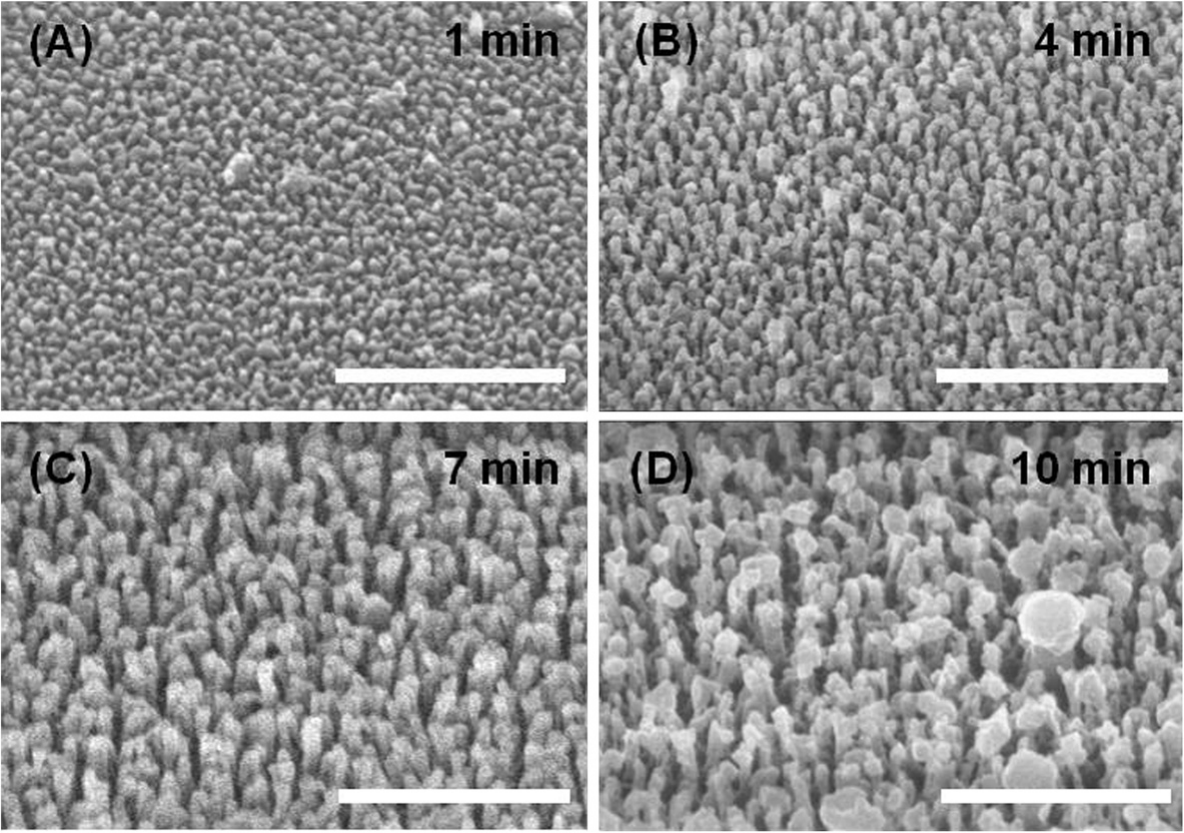
**SEM morphologies of the grassy surfaces fabricated by self-masked etch.** SEM morphologies of the grassy surfaces fabricated by the self-masked etch process of glass substrates with etch times of **(A)** 1, **(B)** 4, **(C)** 7, and **(D)** 10 min, respectively. Scale bar, 1 μm.

**Figure 5:**
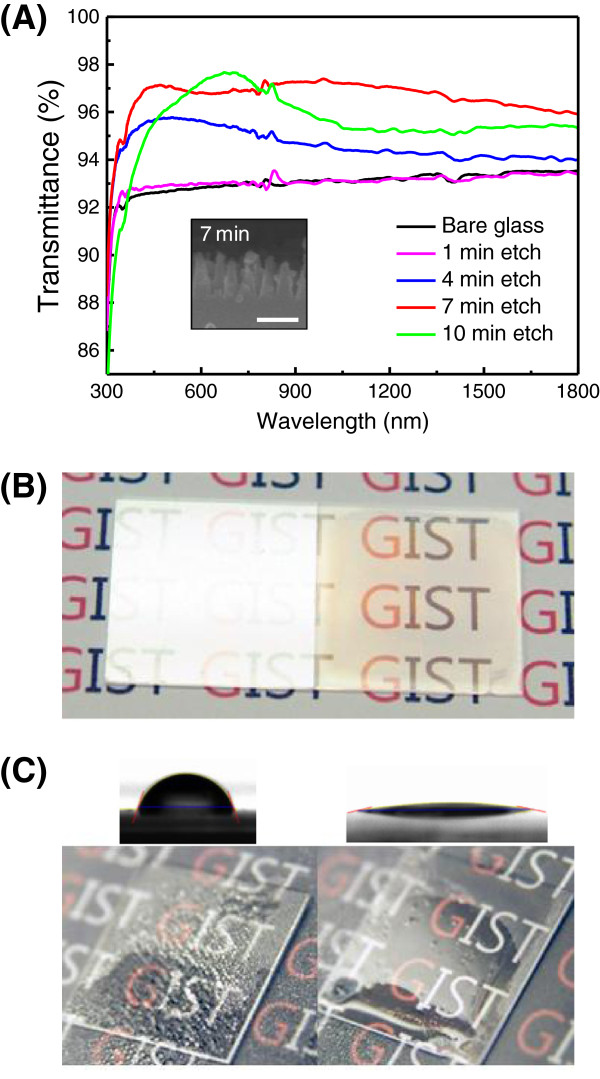
**Transmittance of UV to NIR light and pictures of flat glass and AR glass. (A)** Transmittance of UV to NIR light through a flat reference glass (black solid line) and AR glasses with four different grassy surfaces on both sides. Inset: cross-section SEM image of grassy nanostructures with 7 min etch time. **(B)** Picture of a flat glass (left) and an AR glass (right) with bright illumination light. **(C)** Wetting behavior of the corresponding samples of **(B)**. Inset: contact angle measurement results.

The SEM images in Figure 4 show that grassy surfaces were successfully fabricated using self-masked etch process with a RF power of 50 W. The resulting surfaces are uniform and the average distance between neighboring SWSs are sufficiently short to satisfy zeroth order condition. As the etching time increases, the height of SWSs increases vertically, whereas the density of SWSs decreases because the adjacent structures clumped with each other. This tendency is directly related with the optical behaviors. Figure 5A presents the transmittance curves of glasses with flat and grassy surfaces on both sides in the wavelength range of 300 to 1,800 nm. The glass with flat surface has a transmittance of approximately 93%, which increases monotonically due to the material dispersion. The grassy surface with 1-min etch time has very similar curves with that of the flat surface because the height of grasses is very short. However, the AR effects can be found in all the other grassy surfaces (with 4, 7, and 10 min etch times). After a 7-min etching, the resulting grassy structure has heights of approximately 150 to 200 nm, as shown in the inset of Figure 5A. The average transmittance of glass with grassy surfaces on both sides for 7-min etch time is 96.89% in the visible spectrum (390 to 700 nm), which is 4.15% higher than that of flat surfaces (92.74%). In particular, this high transmittance is sustained over the UV-vis-NIR ranges (i.e., *T*_ave@300–1,800 nm_ = 96.64%). These broadband AR characteristics afford a possibility of the use of this AR glass as a substrate or a cover glass for photovoltaic applications. In case of glass with a 10-min etching, the antireflective property seems to increase from 600 to 900 nm while the broadband AR property is degraded. One of the possible causes on this detrimental change is the reduced density of grassy nanostructures compared to that of glass with a 7-min etching. It is needed to conduct more systematic characterization/analysis to figure out the effect of size, density, and shape of randomly distributed nanostructures on optical properties.

The reflectance difference between the glasses with flat and grassy surface is revealed visually in Figure 5B. An intense light reflection from the flat glass is observed and as a result, reflections occurring at both sides of the glass make the words difficult to read. The grassy surface showed improved readability due to the reduced reflection. In addition to the AR property, the wetting property is also affected by both the structured surface [18] and the oxygen plasma treatment. To confirm the antifogging performance, the SWS-integrated glass and the bare glass were exposed to steam at the same time. Figure 5C shows the antifogging behavior of the glasses with flat and grassy surface. The water droplets beaded up on the flat surface of the bare glass substrate and the bead-like water droplets caused light scattering, which degrades the readability of the words. However, the water droplets on the roughened surface of the SWS-integrated glass evenly spread over the whole surface, and the hydrophilic glass still remained transparent, and the words below it were clearly readable. Water contact angle measurement results also support this hydrophilic effect. The contact angles of glass with and without grassy surface were 12.5° and 71.5°, respectively. The surface energy of structured glass was 87.8 mN/m, which is a higher value than that of bare glass (39.0 mN/m).

## Conclusions

In summary, we demonstrated the subwavelength scale grassy surfaces on the glass substrate by using simple one-step dry etch process without any lithography. The resulting grassy surface showed very high transmittance in very wide spectral ranges as well as antifogging effects. Optimization of self-masked dry etching for improving the optical/material properties remains as a future work. We expect that this low-cost, high-performance optical materials are applicable in various optical and optoelectronic devices.

## Competing interests

The authors declare that they have no competing interests.

## Authors’ contributions

YMS carried out most of the theoretical and experimental works associated with fabrication and characterization of samples, analyzed the results, and prepared the manuscript. GCP and EKK helped the characterization of samples and experimental works. CIY helped the characterization of samples and preparing the manuscript. YTL developed the conceptual framework and supervised the whole work, and finalized the manuscript. All authors read and approved the final manuscript.
